# Molecular Genetics of GLUT1DS Italian Pediatric Cohort: 10 Novel Disease-Related Variants and Structural Analysis

**DOI:** 10.3390/ijms232113560

**Published:** 2022-11-04

**Authors:** Alessia Mauri, Alessandra Duse, Giacomo Palm, Roberto Previtali, Stefania Maria Bova, Sara Olivotto, Sara Benedetti, Francesca Coscia, Pierangelo Veggiotti, Cristina Cereda

**Affiliations:** 1 Department of Biomedical and Clinical Sciences, University of Milan, 20157 Milan, Italy; 2 Newborn Screening and Genetic Metabolic Diseases Unit, V. Buzzi Children’s Hospital, 20154 Milan, Italy; 3 Pediatric Neurology Unit, V. Buzzi Children’s Hospital, 20154 Milan, Italy; 4 Structural Biology Center, Human Technopole, 20157 Milan, Italy

**Keywords:** GLUT1 deficiency syndrome, novel *SLC2A1* variants, GLUT1 structure analysis

## Abstract

GLUT1 deficiency syndrome (GLUT1DS1; OMIM #606777) is a rare genetic metabolic disease, characterized by infantile-onset epileptic encephalopathy, global developmental delay, progressive microcephaly, and movement disorders (e.g., spasticity and dystonia). It is caused by heterozygous mutations in the *SLC2A1* gene, which encodes the GLUT1 protein, a glucose transporter across the blood-brain barrier (BBB). Most commonly, these variants arise de novo resulting in sporadic cases, although several familial cases with AD inheritance pattern have been described. Twenty-seven Italian pediatric patients, clinically suspect of GLUT1DS from both sporadic and familial cases, have been enrolled. We detected by trios sequencing analysis 25 different variants causing GLUT1DS. Of these, 40% of the identified variants (10 out of 25) had never been reported before, including missense, frameshift, and splice site variants. Their structural mapping on the X-ray structure of GLUT1 strongly suggested the potential pathogenic effects of these novel disease-related mutations, broadening the genotypic spectrum heterogeneity found in the *SLC2A1* gene. Moreover, 24% is located in a vulnerable region of the GLUT1 protein that involves transmembrane 4 and 5 helices encoded by exon 4, confirming a mutational hotspot in the *SLC2A1* gene. Lastly, we investigated possible correlations between mutation type and clinical and biochemical data observed in our GLUT1DS cohort, revealing that splice site and frameshift variants are related to a more severe phenotype and low CSF parameters.

## 1. Introduction

Glucose transporter-1 (GLUT1) deficiency syndrome (GLUT1DS), also known as De Vivo disease, is a rare genetic metabolic condition characterized by infantile-onset epileptic encephalopathy, global developmental delay, drug-resistant seizures, progressive microcephaly, and movement disorders (e.g., spasticity, and dystonia) (GLUT1DS1; OMIM #606777) [1,2,3]. Although the infantile-onset epileptic encephalopathy phenotype is the most reported presentation (∼90% of affected individuals), some individuals develop atypical or mild phenotype (∼10%), characterized by infantile-onset paroxysmal exercise-induced dyskinesia (PED) with or without seizures (GLUT1DS2; OMIM #612126) [4,5,6]. Other paroxysmal events, which may be triggered by prolonged exercise, fasting, or emotional stress, include sleep disturbance (e.g., sleep apnea), abnormal eye-head movements, and weakness/paralysis [7,8,9,10]. Low cerebrospinal fluid (CSF) glucose values (termed hypoglycorrhachia) in the absence of hypoglycemia, in combination with low-normal or abnormally low CSF lactate values represent the metabolic hallmark of GLUT1DS [11,12]. These patients are treated with ketogenic diet therapies (KDT) that provide an alternative fuel source for brain energy metabolism12. Effectiveness of the KDT has been observed by Leen et al., not only in patients with drug-resistant epilepsy, but also in patients with movement disorders without epilepsy [5,13,14,15]

The epidemiological data of GLUT1DS are limited and only about 600 cases have been reported in medical literature since 1991 [1]. An incidence of 1.65–2.22 per 100.000 births was recently estimated [16].

GLUT1DS is caused by heterozygous mutations (or rarely, homozygous mutations) in the solute carrier family 2 member 1 gene (*SLC2A1*, 1p34.2) [17,18,19]. The *SLC2A1* gene encodes the glucose transporter-1 (GLUT1). It belongs to the GLUT protein superfamily, composed of 14 members (GLUT1-14) facilitative membrane transporters, involved in the regulation of glucose delivery and metabolism [20]. All GLUT transporters share a common protein structure characterized by 12 α-helices transmembrane (TMH) domains, separated by a large intracellular (IC) loop between helices 6 and 7. The GLUT1 protein is primarily expressed in erythrocytes, in endothelial cells comprising the blood-brain barrier (BBB) and in astrocytes. GLUT1 facilitates glucose diffusion from the bloodstream across the BBB and astrocyte plasma membrane to the central nervous system, representing the fundamental vehicle by which glucose enters the brain [12]. Consequently, a genetic defect in this transporter would impair glucose supply to the brain, resulting in a cerebral energy deficiency that is likely accounting for the clinical manifestations of the GLUT1DS [17,21]. A 3D model for GLUT1 protein has been proposed, predicting a central aqueous channel communicating the intra- and extracellular environment with many amino acid residues crucial for glucose transport located around this central channel [22,23].

So far, a total of 240 pathogenic *SLC2A1* variants have been reported in GLUT1DS patients including deletions, missense, nonsense, frameshift, and splice site mutations (ClinicalStatistics.Varsome.com) [5,24,25]. Most commonly, these mutations arise de novo resulting in sporadic cases, although several familial cases with autosomal dominant inheritance pattern have been reported [25,26]. All these observed mutations are considered to lead to GLUT1DS by a loss of GLUT1 function. Nonsense, frameshift, and splice-sites *SLC2A1* variations resulting in 50% loss of GLUT1 protein are often associated with the early-onset classical phenotype, whereas mild or moderate forms of the disease are most likely associated with missense *SLC2A1* mutations resulting in 50–70% residual function of GLUT1 [5,15,24,27]. However, genotype-phenotype correlation remained elusive with high interindividual phenotypic variability, even between mutated members of the same family [15,24].

The purpose of the study was to investigate the molecular data of a large Italian cohort of 27 pediatric patients clinically suspect of GLUT1DS from both sporadic and familial cases; specifically, we performed trios sequencing analysis, highlighted 10 novel disease-related mutations and their pathogenic effects by X-ray structure analyses, and then investigated possible correlations between mutation type and clinical and biochemical data.

## 2. Results

### 2.1. Clinical and Biochemical Features

The main clinical and biochemical characteristics of 27 patients are shown in Table 1.

The most frequent phenotype (*n* = 23; 85.2%) represented the epilepsy. Among these 23 patients, 17 had an onset of seizures before the age of two years (early-onset classical phenotype; 73.9%), while in six patients the onset of seizures occurred later in life (late-onset classical phenotype; 22.7%). Seizure semiology was diverse (tonic-clonic, dyscognitive, epileptic spasms), and also the frequency varied from daily to sporadic. Intellectual disability (ID) ranged from mild (*n* = 4; 23.5%) to severe (*n* = 7; 41.1%) in patients with the early-onset classical phenotype (two patients had moderate ID, one borderline ID and two normal ID, while data of one patient were unavailable); in six patients with late-onset classical phenotype, three (50%) patients had severe ID, two (33.3%) mild ID, and one patient had normal ID. Movement disorders (ataxia, dystonia, choreoathetosis) are shown in 73.9% (*n* = 17) out of 23 of the patients with seizures (13 patients with the early-onset and four in late-onset classical phenotype). Microcephaly (head circumference less than 3rd percentile for age and sex) occurred in one of 17 patients with the early-onset classical phenotype (patient 3).

Most of these patients did not respond to antiepileptic drugs, such as phenobarbital, and therefore they were treated with KDT (*n* = 19; 70.3%). Among the patients treated with ketogenic diet, 17 out of 19 (94.4%) presented with epilepsy. In the epilepsy group, 14 out of 17 (82.4%) individuals fully responded and are now seizure free. In two cases (11.8%) there were a mild response with some degree of seizures reduction, without reaching seizure freedom; and one patient did not present seizure frequency improvement. For patients who achieved seizure freedom with KDT, the treatment did not significantly influence movement disorders nor lead to an improvement in the intelligence quotient.

Furthermore, the non-classical phenotype characterized by paroxysmal exercise-induced dyskinesia without seizures occurred in three out of 27 patients (11.1%). One of these three patients presented microcephaly (patient 20).

Finally, patient 19 presented only minimal symptoms: mild intellectual disability and microcephaly.

CSF/blood glucose ratios (reference range 0.6 mg/dL) in all patients ranged from 0.25 to 0.52 (mean 0.39; SD 0.06) (data of patient 16 and patient 23 were unavailable).

### 2.2. Molecular Data

**Figure 1:**
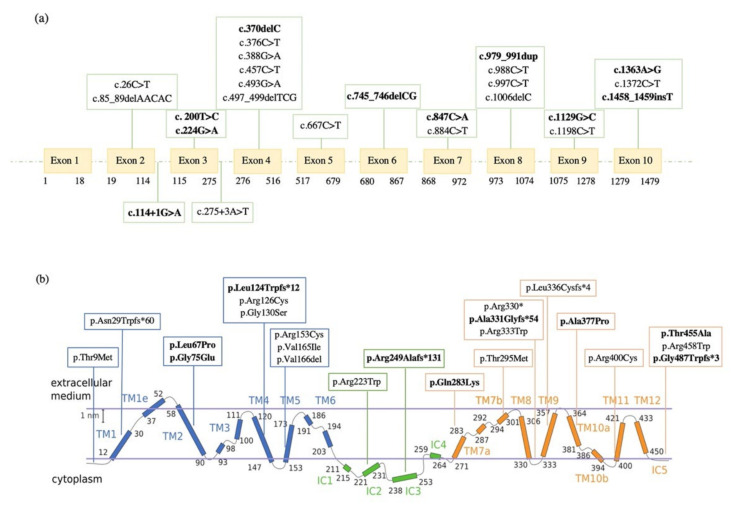
(**a**) Distribution of 25 different variants in the *SLC2A1* gene identified in 27 patients with GLUT1DS. (**b**) Representation of the GLUT1 protein domains and mapping of 25 different mutations reported in our GLUT1DS patients (adapted from Galochkina et al., 2019 [23]. More details on licensing are available via the following link http://creativecommons.org/licenses/by/4.0/, accessed on 28 October 2022). Novel variants identified in our cohort are reported in bold.

Sequencing analysis of the 27 patients detected 25 different variants in the *SLC2A1* gene (Figure 1a), including 15 missense (60%), six frameshift (24%), one nonsense (4%), one splice site (4%), one in-frame (4%) and one noncoding (4%) (Table 2). Of these, 10 mutations (40%) have never been described before. Moreover, 24% (six out of 25 identified variants) is located on transmembrane helix 4 (TMH4) and transmembrane helix 5 (TMH5) encoded by exon 4, confirming a mutational hotspot in the *SLC2A1* gene observed by Pascual et al. [28].

**Figure 2:**
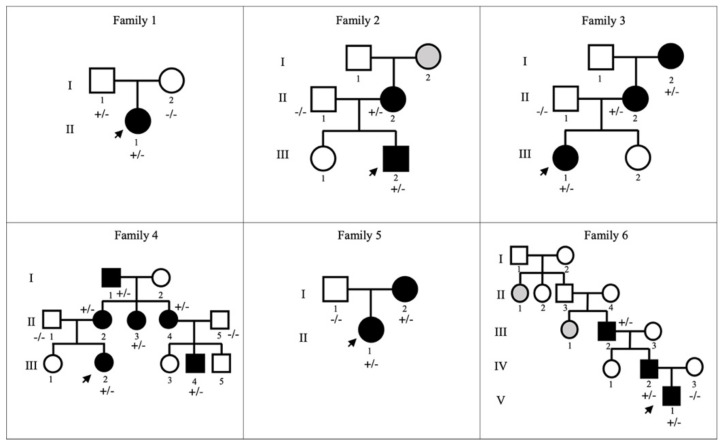
Pedigree of the familial cases with GLUT1DS. In family 2 and family 6, grey fills denote patients with clinical phenotype but unavailable for the study.

Among the GLUT1DS pediatric patients, 21 mutations occurred de novo, resulting in sporadic cases, while six were familial cases with clinically and genetically affected relatives, bringing the total number of individuals with *SLC2A1* variants to 39. The pedigrees of the six families with autosomal dominant transmission are reported in Figure 2.

According to the ACMG criteria, 16 variants were classified as pathogenic, five variants reported as likely pathogenic and four variants of uncertain significance (VUS). Although these four variants (c.26C > T, c. 200T > C, c.275+3A > T and c.1363A > G) are classified as VUS, they were considered possibly causative based on the clinical phenotype of the patients, in silico predictions and/or family segregation.

### 2.3. Genetic Variations in Familial Cases

We identified six families with autosomal dominant transmission of GLUT1DS.

Family 1. The proband (II-1; patient 1) was a 16-year-old girl presenting recurrent absence seizures since the age of six without movement disorder episodes. She was treated with different antiseizure medications, with no improvement in her condition. Her parents (I-1, I-2) are reported to be in good health.

Genetic analyses performed in the family trio revealed the presence of the heterozygous missense variant c.26C > T (p.Thr9Met) in exon 2 of the *SLC2A1* gene in the proband (II-1), inherited from the unaffected father (I-1). To determine the impact of this amino acid substitution in position 9, the pathogenic score was evaluated by 19 different *in silico* predictors, and it was predicted to be damaging by 17 of them (Table S1). This score is consistent with the amino acid change from a polar threonine to a non-polar methionine, in a highly conserved residue. The identified variant (rs1570601100) is classified as VUS in ClinVar (SCV000953099), reported as likely pathogenic in LOVD database and as a likely disease-causing mutation for GLUT1DS by HGMD Professional 2021.3. This same variant (c.26C > T) has been reported, for the first time, by Castellotti et al. in an individual affected with GLUT1DS and, as in our family 1, also in the asymptomatic father [29].

Family 2. The proband (III-2, patient 4) of this family was a 16-year-old boy suffering recurrent uncontrolled lower limb movement since the age of seven; free periods of six months were intercalated by weeks in which PED occurred every day (after intense exercise, or at the end of a stressful day). At the age of two, the patient had two absence-like-episodes, not related to fever. The mother (II-2), a 49-year woman, suffered from recurrent episodes of paroxysmal exercise induced dyskinesia during adolescence (since 13 years old), then spontaneous resolution occurred in her early twenties. Moreover, the family reported seizures in maternal grandmother (I-2, not available for sequencing). 

Trio sequencing analyses identified the novel heterozygous missense variant c.200T > C (p.Leu67Pro) in exon 3 of the *SLC2A1* gene in the proband (III-2), inherited from the affected mother (II-2). This amino acid change from leucine to proline (p.Leu67Pro) affects a highly conserved on transmembrane helix 2 (TMH2), likely disrupting the domain’s α-helical architecture. The pathogenic score, indeed, was evaluated by 19 different *in silico* predictors and it was predicted to be damaging by 18 of them (Table S1). The *SLC2A1* variant identified in this family is not present neither in ClinVar nor in HGMD Professional 2021.3.

Family 3. The proband (III-1, patient 9) was a 9 years old girl with recurrent generalized tonic-clonic seizures and absence seizure since the age of three. With growth, during childhood, she suffered from dyskinesia triggered by exercise or fatigue to the lower limbs. EEG showed slow background activity generalized and focal discharges. The mother (II-2), a 41-year-old woman, affected by generalized tonic-clonic seizures, since the age of two. She suffered from paroxysmal exercise induced dyskinesia, worsened with growth, mainly in stressful periods. Those movements affected, with the same severity and same frequency, both arms and legs. The maternal grandmother (I-2) was a 73 years old woman with several generalized tonic-clonic seizures since the age of 17, maintained under control with a combination of different antiepileptic drugs. Since the age of eight, she also developed PED, which has worsened with growth. The lower limbs, especially her feet, were the most affected. In the last years, these movement disorders became rare, occurring only at the end of intense exercise-fatigue. 

Sequencing analysis allowed the identification of the heterozygous missense variant c.388G > A (p.Gly130Ser) in exon 4 of the *SLC2A1* gene in the proband (III-1), in the mother (II-2) and in the grandmother (I-2). To determine the impact of this amino acid change, the pathogenic score was evaluated by 19 different *in silico* predictors, and it was predicted to be damaging by all of them (Table S1). The Gly130Ser mutation occurs at the narrow interface between TMH4 and TMH2, replacing a glycine, which lacks a side chain, with a cysteine whose side chain could sterically clash with TMH2, thereby destabilizing the helical packing of the transporter (Figure S1b). The identified variant (rs80359819) is classified as pathogenic in ClinVar (RCV000770978), as likely pathogenic in LOVD database, and as a disease-causing mutation for GLUT1DS by HGMD Professional 2021.3. It has been previously identified in GLUT1DS patients [24].

Family 4. The proband (III-2, patient 11) of this family was a 17 years old girl with no seizure history, but recurrent paroxysmal exercise induced dyskinesia since the age of six. This movement disorder was characterized by worsening due to fatigue and fasting. The mother (II-2), a 38-year woman, affected by generalized tonic-clonic seizure and absence seizure from the age of one, with seizures every two-three days. She was on therapy from the age of one, with a combination of different antiepileptic drugs, with poor outcome. She was also affected by paroxysmal exercise induced dyskinesia since the age 25, with legs more affected than arms. Additionally, a stroke-like episode occurred, with paresis of the upper limb and loss of language functions for 12–24 h. The aunt (II-3) was affected by absence seizures since the age of six. She has not developed PED episodes during her life. The aunt (II-4) of the proband (III-2) was a 34 years old woman who suffered of absence seizures since the age of six, once every two-three weeks. Since the age of 20, she has experienced paroxysmal exercise induced dyskinesia episodes. When she was 31 years old, she had two stroke-like episodes with left hemiparesis. She has three sons, one (III-4, a 7 years old boy) affected by generalized epileptic seizure, mild intellectual disability and attention-deficit/hyperactivity disorder (ADHD); the other one by ADHD, and the last one, apparently, in healthy conditions. The maternal grandfather (I-1) was a 71 years old man; he had recurrent migraines during adolescence, with dizziness just at the end of a physical effort, and PED. No other pathology was referred.

The heterozygous missense variant c.493G > A (p.Val165Ile) in exon 4 of the *SLC2A1* gene was identified in all family members showing epilepsy and/or PED episodes. This amino acid change (p.Val165Ile) affects a highly conserved residue whose position in TMH5 is crucial for glucose transport. The pathogenic score, indeed, was evaluated by 19 different in silico predictors, and it was predicted to be damaging by 15 of them (Table S1). The identified variant (rs1057520545) is classified as pathogenic in ClinVar (SCV002247281) and LOVD database, and as a disease-causing mutation for GLUT1-DS by HGMD Professional 2021.3. This same variant (c.493G > A) has been previously observed in individuals with GLUT1DS [9,36].

Family 5. The proband (II-1, patient 16) was a seven years old girl presenting recurrent absence seizures since the age of six months. Moreover, since the age of two, she is affected by exercise-induced dyskinesia. The mother (I-2), a 40 years old woman, affected by seizure since childhood. She suffered from paroxysmal exercise induced dyskinesia episodes, mainly in stressful periods. She had a stroke episode. 

NGS analysis performed in the family trio showed the heterozygous missense variant c.884C > T (p.Thr295Met) in exon 8 of the *SLC2A1* gene in the proband (II-1), inherited from the affected mother (I-2). To determine the impact of this amino acid substitution in position 295, the pathogenic score was evaluated by 19 different in silico predictors, and it was predicted to be damaging by 18 of them (Table S1). This score is consistent with the amino acid substitution from a polar threonine to a non-polar methionine, in a highly conserved domain (TMH7) comprising the GLUT1 cavity. This is also supported by functional studies on this variant [37]; in particular, the authors showed that this amino acid change (p.Thr295Met) specifically alters the GLUT1 conformation and affects the glucose uptake. The rs80359823 variant is classified as pathogenic in ClinVar (SCV001577959) and LOVD database, and as a disease-causing mutation for GLUT1DS by HGMD Professional 2021.3, as it has been previously identified in GLUT1DS patients [24]. This same *SLC2A1* variant (c.884C > T) was also showed in patient 17, where both parents had been tested, as a de novo mutation, resulting in a sporadic case.

Family 6. The proband (V-1, patient 25) was a three years old boy. Since the age of four months, he suffered from several partial seizures, characterized impaired awareness, cyanosis, apnea, and tonic spasms. Frequent events in the same day (9–10 times in a day) were interrupted by seizure-free periods. The duration of each episode ranged from 10 s up to 30–40 s. The father (IV-1) suffered of paroxysmal exercise induced dyskinesia since the age of 10. The most affected limbs were the legs, and the episode duration was related to the exercise intensity. During adolescence those episodes worsened in terms of frequency and intensity, occurring daily and in his twenties, these episodes became less frequent. The paternal grandfather (III-2) showed recurrent PED, right lower limb was mainly affected (rarely arms were involved). It started at the age of 12, with increased intensity and frequency up to 20 years old, mainly 1–2 times in a month. These abnormal movements were mainly associated with stressful periods or right after physical exercise. Nowadays, the only remaining symptom are the migraine episodes that have been occurring throughout his life, starting in adolescence. Symptoms also occurred in other family members: sister of patient III-2 (III-1) was affected by recurrent and severe seizures, and anxiety disorder; the aunt of patient III-1 and III-2 (II-1) was affected by seizure and movement disorder (II-1, III-1 not available for sequencing).

The novel heterozygous missense variant c.1363A > G (p.Thr455Ala) in exon 10 of the *SLC2A1* gene was identified in the proband (IIIII-1), in the father (IIII-2) and in the grandfather (III-2). The *SLC2A1* variant identified in this family is neither classified in ClinVar nor in HGMD Professional 2021.3. To determine the impact of this amino acid substitution (p.Thr455Ala), the pathogenic score was evaluated by 19 different in silico predictors, and it was predicted to be damaging by 18 of them (Table S1). This score is consistent with the amino acid change in position 455 from a polar threonine to a non-polar alanine, in a highly conserved residue of IC5 involved in the GLUT1 cavity.

### 2.4. Genetic Variations in Sporadic Cases

Whereas in all familial cases (100%) we detected missense mutations, only 52.4% (11 out of 21) of sporadic cases had missense variants; in the remaining 10 patients with de novo mutations, we identified frameshift (28.6%) and nonsense, splice site, in-frame, and noncoding variants (19%). We found 14 pathogenic variants, six likely pathogenic and one VUS, according to ACMG criteria.

To note, the variant classified as uncertain significance was a heterozygous de novo noncoding mutation c.275+3A > T detected in patient 6. It has been previously identified in GLUT1DS patient by Graziola et al. [30], and considered causative, as in our case, based on clinical presentation of the proband. Further in silico analysis on Genomnis HSF suggested that this variant (c.275+3A > T) most probably affected the splicing process, leading to alteration of the 5′ splice site in intron 3.

### 2.5. Novel SLC2A1 Variants and Their Crystallographic Structure

Out of a total of 25 identified variants, 40% have never been reported before (eight de novo and two inherited), including one splice site (10%), four frame shift (40%), and five missense (50%).

**Figure 3:**
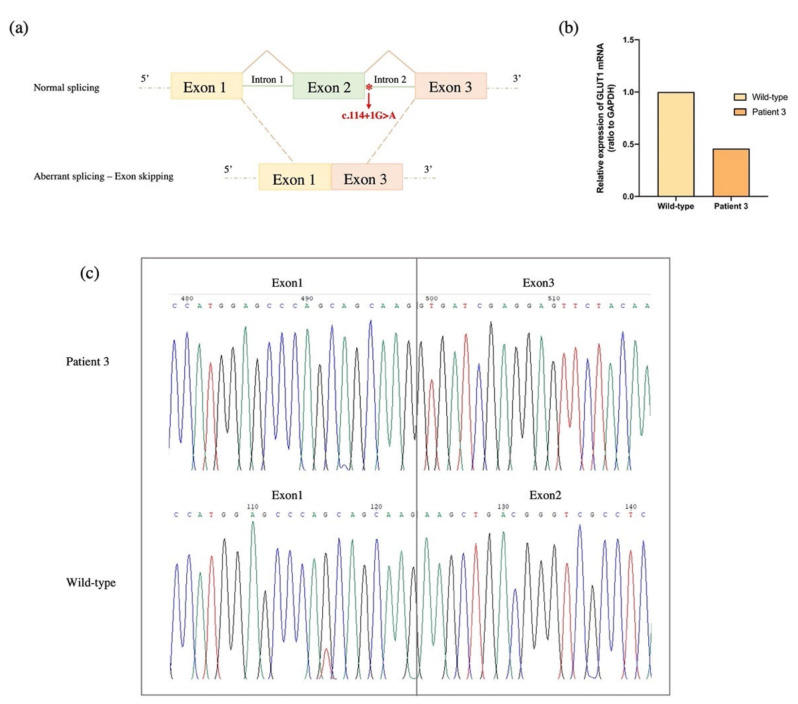
(**a**) Schematic illustration of normal (above) and aberrant splicing (bottom), which results in exon 2 skipping. The red asterisk indicates the novel heterozygous de novo c.114+1G > A variant in intron 2. (**b**) Relative expression analysis of GLUT1 alternative isoform by RT-qPCR in the patient 3. GLUT1 expression data are normalized on GAPDH expression levels. (**c**) Sequence electropherograms showing the GLUT1 transcript with skipping of exon 2, resulting from the identified c.114+1G > A mutation, in patient 3 (above). As expected, the full-length GLUT1 transcript was identified in wild-type GLUT1 (bottom).

The novel heterozygous de novo splice site variant c.114+1G > A identified in patient 3 affected the donor splice site of intron 2, possibly leading to exon 2 skipping. This hypothesis was confirmed by the in silico analyses of the splicing process of the alternative transcript. Splicing prediction tools (e.g., Fruit Fly Splice Predictor, ESEfinder 3.0, Genomnis HSF), indeed, suggested that the novel variant c.114+1G > A causes the loss of the 5′ splice site in intron 2 and the consequent skipping of exon 2, thus leading to exon 1–3 junction (Figure 3a). In particular, the in-frame loss of exon 2 causes the completely loss of the first transmembrane helix (TMH1) of the GLUT1 (predicted by protein topology THMM in silico tool), which is highly involved in glucose uptake, probably due to its important role for the protein state transition [23]. To confirm in silico prediction, we performed GLUT1 transcript analysis on the RNA samples from blood of patient 3 and wild-type GLUT1. RT-qPCR (Figure 3b) and Sanger sequencing (Figure 3c) confirmed that the identified mutation leads to the formation of an aberrant GLUT1 transcript with skipping of exon 2 (GLUT1 skipped) (Figure 3c). Patient 3 shows, indeed, the expression of this alternative transcript, while the wild-type, as expected, presents the GLUT1 full-length.

The novel frameshift mutations identified in our patients cause the introduction of a PTC, predicted to lead to the synthesis of non-functional truncated protein, or more likely to NMD. It is likely that these mutations result in 50% loss of GLUT1 protein and thus, lead to severe impairment of glucose transport into brain. These molecular findings were also supported by both biochemical and clinical data of patients 7, 14, 18, and 27: the CSF/blood glucose ratios were 0.36, 0.37, 0.31, and 0.37 mg/dL, respectively; all patients had the early-onset severe phenotype in combination with intellectual disability and/or movement disorders.

**Figure 4:**
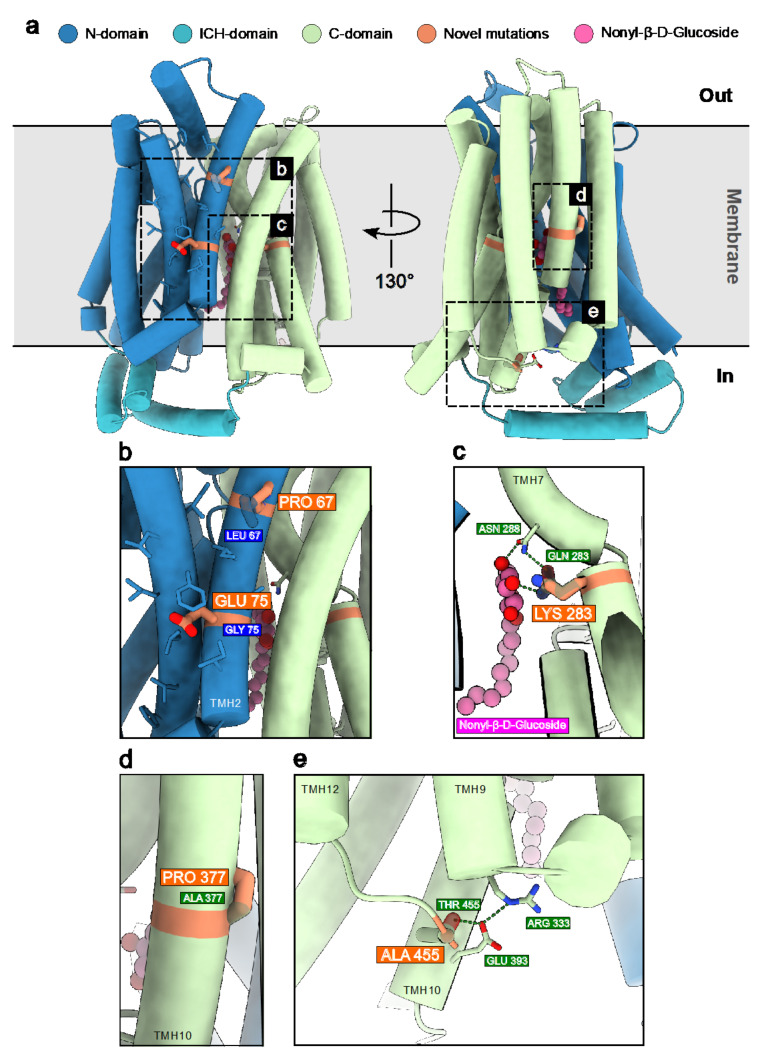
Novel disease-related mutations mapped on the structure of GLUT1. (**a**) X-ray structure of human GLUT1 (PDB ID: 6THA) bound to Nonyl-β-D-Glucoside via its glucose head group. Novel disease-related mutations identified in this study are depicted in orange and superimposed on their corresponding wild-type residues. (**b**), Mutations Leu67Pro and Gly75Glu on transmembrane helix 2 (TMH2) could disrupt the α-helical architecture and the hydrophobicity of the region, respectively. (**c**) Gln283Lys could hamper the hydrogen bond network that stabilizes glucose binding. (**d**) Ala377Pro could destabilize the α-helical architecture of TMH10. (**e**). Thr455Ala potentially breaks the hydrogen bond network between residues on TMH9, TMH10 and TMH12, possibly reducing the stability and rigidity of the C-terminal domain of GLUT1.

The amino acid changes in the novel missense variants identified in this study were evaluated by nineteen different in silico predictors, and they were predicted to be damaging by most of them (Tables S1 and S2). Additionally, in order to rationalize how these novel disease-related missense mutations may impact the structure and function of human GLUT1 (hGLUT1), we mapped them on its X-ray crystallographic structure in complex with Nonyl-β-D-Glucoside [38,39] (PDB ID: 6THA) (Figure 4a). The latter is a detergent bearing a glucoside group which binding to the hGLUT1 pocket that in other structures of hGLUT1 homologues is occupied by the native ligand glucose. For example, in hGLUT3 structure, PDB ID: 4ZW9 [40] and the *E. coli* homolog of GLUT1-4, XylE, PDB ID: 4GBZ [41]. Similarly, in another equivalent reported structure of hGLUT1, the same pocket is occupied by the cytochalasin B inhibitor (PDB ID: 5EQI) [42]. As follows, we discuss the potential significance of each newly identified variant in the context of their chemical environment and position in key secondary structure elements of hGLUT1 (Figure 4b–e). The Leu67Pro mutation, located on TMH2, may cause a kink of the α-helix due to the rigidity of the cyclic pyrrolidine side chain of proline (Figure 4b). Despite not being directly involved in ligand binding, this mutation could affect the overall protein helix bundle flexibility and therefore the glucose transport efficiency. The Gly75Glu variant, also found in TMH2, corresponds to the replacement of a small hydrophobic glycine residue with a negatively charged glutamic acid on the membrane-facing side of TMH2 (Figure 4b). Here, the mutation could in principle affect not only the folding, but also the localization of hGLUT1 in the phospholipidic membrane. Gln283Lys is found right in the internal cavity of the hGLUT1 transporter where glucose transits (Figure 4c). Here, the WT residue Gln283 forms a hydrogen bond network with Asn288, likely involved in stabilizing the glucose head group of the Nonyl-β-D-Glucoside, mimicking the natural ligand. The replacement with a longer and charged residue as lysine may likely disrupt this highly specific binding configuration, probably modifying the glucose passage through the channel. Moving to a different region of the transporter, the Ala377Pro mutation located on the transmembrane helix 10 (TMH10) could potentially have a similar destabilizing effect as the Leu67Pro mutation in TMH2 (Figure 4d). The Thr455Ala mutation is located within the C-terminal portion of hGLUT1 in a flexible hinge after TMH12 (Figure 4e). Interestingly, Thr455 establishes a network of interactions with Glu393 on TMH10 and with Arg333 on TMH9, holding the three C-terminal α-helices together. These may also explain why Thr455 is the last semi-rigid and visible residue in the crystallographic model. The pathogenicity of the Thr455 to alanine mutation could probably be related to the disruption of this C-terminal “lock” network with a detrimental increase on the C-terminal domain flexibility and possibly on the dynamics of ligand transport.

### 2.6. Genotype, Phenotype and Biochemical Correlations

Given the clinical variability and the wide spectrum of heterozygous mutations (including missense, frameshift, nonsense, and splice site variants) observed in our study cohort, we investigated possible associations between phenotype, genotype, and biochemical data.

**Figure 5:**
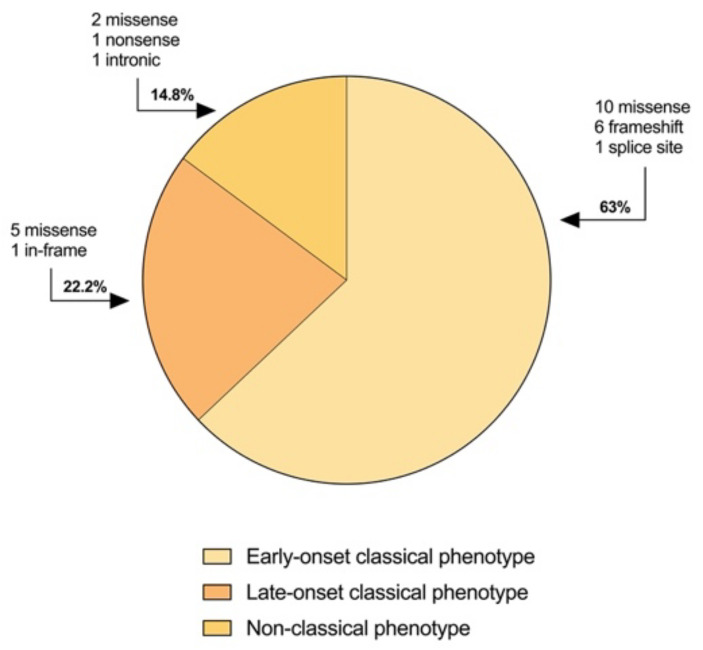
Distribution of the 27 *SLC2A1* variants observed in our cohort according to the different phenotype.

The early-onset classical phenotype was seen in both patients with missense mutations (59%; *n* = 10) and patients with splice site and frameshift variants (41%; *n* = 7). All the patients with splice site and frameshift variants had the early-onset classical features, whereas 41% (*n* = 7) of the patients with missense mutations had the late-onset or non-classical phenotype (Figure 5). Patients with identical missense mutations (16, 17, and 24, 25) were heterogeneous in clinical manifestations.

**Figure 6:**
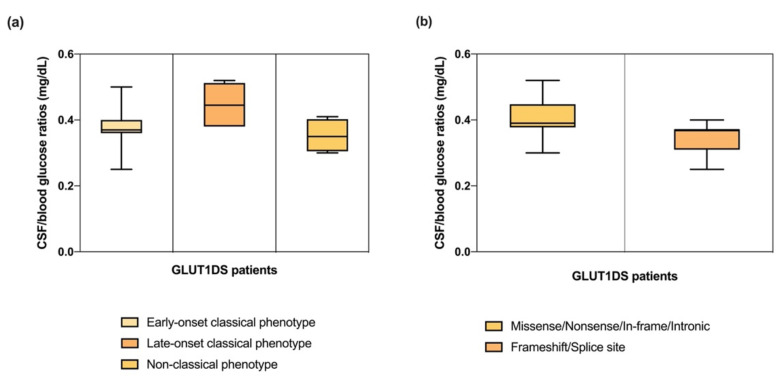
Genotype, phenotype, and biochemical correlations. (**a**) Boxplot shows CSF/blood glucose ratios with three different phenotypes. (**b**) Boxplot displays CSF/blood glucose ratios with different type of variants.

Patients with the late-onset classical phenotype had a higher CSF/blood glucose ratio (mean 0.44; SD 0.07) than patients with the early-onset classical phenotype (mean 0.37; SD 0.05) and patients with non-classical clinical features with movement disorders without epilepsy (mean 0.35; SD 0.05) (Figure 6a).

The CSF/blood glucose values were lower in patients with splice site and frameshift mutations (mean 0.34; SD 0.05) than in individuals with missense mutations (mean 0.40; SD 0.05) (Figure 6b).

## 3. Discussion

For the first time, we reported the molecular data of 27 Italian children with a clinical suspect of GLUT1DS; specifically, we performed *SLC2A1* sequencing analysis, investigated possible correlations between phenotype, genotype and biochemical data and then highlighted novel disease-related mutations and a potential mutational hotspot in exon 4 of the *SLC2A1* gene.

In our cohort we have identified 17 patients (62.9%) with the early-onset classical phenotype, five patients (18.5%) with the late-onset classical phenotype, while in the remaining four patients (14.8%) we observed a non-classical phenotype of movement disorders without epilepsy. This latter phenotype is considered uncommon and has only been described in a small number of patients [12,14]. We have, however, identified in patient 19 only minimal symptoms such as intellectual disability and microcephaly, suggesting a wide clinical spectrum of GLUT1 deficiency syndrome.

CSF/blood glucose ratios were below 0.52 mg/dL in all our patients, and below 0.37 mg/dL in patients with splice site and frameshift mutations (25.9%), as showed in Figure 6b, and thus suggesting that genotype could be correlated with CSF parameters. All these mutations, resulting in 50% loss of GLUT1 activity, are identified in patients with the early-onset classical phenotype in combination with intellectual disability and/or movement disorders (patients 2, 3, 7, 14, 18, 21, 27). This highlights that low CSF/blood glucose ratios are most likely related to a more severe phenotype, as well as with splice site and frameshift variants.

Our molecular findings showed important differences between sporadic and familial cases in the type of mutations, and thus in the severity of the clinical manifestations. Heterozygous missense variants detected in all familial cases, as is now well established [43], were associated with mild to moderate forms of the disease with broad phenotypic variability: the early-onset classical phenotype was observed in patients 9 (family 3), 16 (family 5), and 25 (family 6), the late-onset was noted only in patient 1 (family 1), whereas patients 4 (family 2) and 11 (family 4) presented non-classical features with movement disorders without epilepsy. Furthermore, we observed wide clinical heterogeneity even within the same family [44]. Patients with the same mutation (c.493G > A) in family 4 displayed a heterogenous range of type and severity of GLUT1DS phenotype. In family 1 the identified c.26C > T variant is present in the affected proband (patient 1) and in the unaffected father. This phenotypic diversity within families with autosomal dominant transmission of GLUT1DS suggests a modulating effect of DNA regulatory elements of the *SLC2A1* gene that may modulate the expression level of the GLUT1 protein. This assumption emphasizes the importance of further clinical and genetic/epigenetics investigation of GLUT1DS families.

Overall, 40% of the identified variants in our *SLC2A1* cohort (10 out of 25) had never been reported before, including missense, frameshift, and splice site variants, broadening the genotypic spectrum heterogeneity found in the *SLC2A1* gene. Although two of these (c.200T > C and c.1363A > G) were classified as VUS according to the ACMG criteria, X-ray structure analyses of GLUT1 strongly suggested the potential pathogenic nature of these novel variants (Figure 4), providing additional evidence to overcome the challenging VUS classification. Instead, the pathogenicity of the two VUS identified in patient 1 and patient 6 was supported by literature data [29,30].

A significant fraction (six out of 25; 24%) of the identified variants is located in a vulnerable region of the GLUT1 protein that involves TMH4 and TMH5 domains encoded by exon 4, as showed in Figure 1b and represented on the same reference hGLUT1 structure in Figure S1. TMH4 and TMH5 include mutations at amino acid residues Leu124, Arg126, Gly130, Arg153, Val165, and Val166, which are located around GLUT1 central channel and involved in the interaction with the glucose. During the protein state transition for the glucose uptake, many residues, including Arg126 on TMH4, form strong hydrogen bonds with the glucose molecules. Of note, the amino acid position 126 is exposed at the extracellular part of the GLUT1 protein and a positively charged arginine facilitates glucose transport, resulting in a glucose binding site residue [23]. Thus, the amino acid change from a positively charged arginine to a shorter polar side chain of cysteine (p.Arg126Cys) observed in patient 8, could affect the binding affinity of GLUT1. At the same time, the disruption of the salt bridge at the intracellular part of the transporter between TMH5, TMH10, and IC3 (Arg153, Glu397, and Glu247), due to the Arg153Cys mutation on TMH5 (detected in patient 10), could lead to the dramatical decrease of the glucose uptake [23]. These molecular findings are also supported by both biochemical and clinical data of patients 8, 9, and 10: the CSF/blood glucose ratios are 0.34, 0.39, and 0.39 mg/dL, respectively. Additionally, stroke episodes occurred in patients 8 and 10, suggesting a critical functional disturbance associated with structural alterations in TMH4 and TMH5 domains of the GLUT1 transporter.

In summary, these molecular data confirmed also in our cohort the mutational hotspot in exon 4 of the *SLC2A1* gene observed by Pascual et al. [28], suggesting that structural alterations in TMH4 and TMH5 domains of the GLUT1 transporter could affect the glucose uptake into the brain. Additionally, our discovery of the 10 novel disease-related variants and their structure analyses broadens the genotypic spectrum heterogeneity found in the *SLC2A1* gene, suggesting the pathogenic effects of these identified mutations. Lastly, the wide clinical and genetic heterogeneity observed in our GLUT1DS cohort allowed possible correlations between mutation type and clinical and biochemical data. This analysis enabled to delineate that splice site and frameshift variants are related to a more severe phenotype characterized by early-onset classical phenotype and low CSF parameters.

## 4. Materials and Methods

### 4.1. Study Population

We enrolled 27 Italian pediatric patients who were referred to the *SLC2A1* gene analysis by the physicians of Buzzi Children’s Hospital in Milan for a clinically suspect of GLUT1DS. For all patients we sequenced trios (proband and parents), and when available other family members (i.e., family 3, family 4, and family 6). The probands include 17 females and 10 males with ages ranging from five months to 19 years (average six years) at the time of recruitment.

This study was approved by the ethics committee of Area 1 of Milan (2021/ST/004) and written informed consent was obtained from all pediatric patients and their family.

### 4.2. CSF Biochemical Analysis

In all patients, cerebrospinal fluid (CSF) was collected through the spinal tap procedure. Then, the quantification of glucose in CSF was performed using the Alinity c Glucose enzymatic assay (Abbott Laboratories).

### 4.3. Mutation Analysis of the SLC2A1 Gene

Genomic DNA from probands and relatives were extracted from peripheral blood leukocytes (PBLs) using a semi-automated method Maxwell System DNA Purification (Promega, Madison, WI, USA), according to the manufacturer’s instructions. The genetic analyses have been performed Next Generation Sequencing (NGS) panels.

NGS analyses were performed using an Agilent custom SureSelect panel (Agilent, Santa Clara, CA, USA) and libraries were sequenced on the Illumina NextSeq 550 platform (Illumina, San Diego, CA, USA). Sequences were aligned to the reference genome (GRCh38), and variants were called through the Sentieon (Sentieon Inc, San José, CA, USA) and annotated using VarSeq (Golden Helix, Bozeman, MT, USA). Reads with low quality calling score and coverage <10, reads falling in segmental duplicated regions (SuperDups > 0.9), and common SNPs (Minor allele Frequency (MAF) > 1%) were filtered and removed.

Sanger sequencing was used to confirm the NGS variations found in patients or to define variations in parents or other family members. We used a standard protocol to amplify all 10 exons of the *SLC2A1* gene using primers located in adjacent intronic regions on gDNA by polymerase chain reaction (PCR). All amplicons were screened for sequence variations by direct sequencing using Big-Dye Terminator v3.1 sequencing kit (Applied Biosystems, Foster City, CA, USA) and ABI 3130 Genetic Analyzer (Applied Biosystems, Foster City, CA, USA). Each fragment was sequenced on both strands. The alignment to the reference sequence (NM_006516) was performed using the Sequencher 4.8 software (Gene Codes Corporation, Ann Arbor, MI, USA).

The identified variants in the *SLC2A1* gene were interpreted according to the American College of Medical Genetics and Genomics (ACMG) guidelines [45] with the support of several tools such as Varsome, Franklin by Genoox (https://franklin.genoox.com (accessed on 5 September 2022)) and locus specific-variant databases (including HGMD and ClinVar). Several in silico prediction tools (e.g., SIFT, PolyPhen2, MutationTaster) were used to assess pathogenicity scores; in particular, we used the MutationTaster tool (https://www.mutationtaster.org (accessed on 12 September 2022) to define the position of premature termination codons (PTCs) compared to the canonical ones and to predict the effect of nonsense-mediated decay (NMD) according to the rules governing this surveillance process [46].

GLUT1 structural analysis related to patient variations in Figure 4 and Figure S1 were performed with UCSF ChimeraX [47]. GLUT1 structural analysis related to patient variations in Figure 4 and Figure S1 were performed with UCSF ChimeraX [47]. The human GLUT1 structure coordinates, as reported in the protein data bank (PDB) with PDB ID: 6THA were represented using the “cylinder display option” for the solute carrier transmembrane helices. The pathogenic aminoacidic variants were then selected, overlapped with the mutated amino acid, and highlighted as sticks. Therefore, by simple structural inspection, depending on the variant amino-acidic nature and position with respect to the substrate or the key folding elements, we discussed their potential implications on GLUT1 stability and glucose binding and transport.

### 4.4. RNA Isolation, RT-qPCR and cDNA Analysis

RNA from PAXgene blood RNA tubes of patient 3 and wild-type GLUT1 was extracted with PAXgene blood RNA kit (Qiagen, Hilden, Germany).

Reverse transcription was performed using the iScript cDNA synthesis Kit (BioRad, Hercules, CA, USA) and the SsoAdvanced^TM^ Universal SYBR^®^ Green Supermix (Bio-Rad, Hercules, CA, USA) was used as dye to perform qPCR on the CFX Connect Real-Time RT-PCR System (Bio-Rad, Hercules, CA, USA), using the following GLUT1 primers F: 5′-TGGCTCCTTCTCTGTGGGCCTT-3′, R: 5′-GGACACGAAGGCCAGCAGGTTC-3′. Gene expression profiling was calculated using the ΔΔCt method, and GAPDH (NM_002046) was used as housekeeping gene for normalization (F: 5′-CTTTTGCGTCGCCAG-3′, R: 5′-TTGATGGCAACAATATCCAC-3′).

cDNAs were amplified and sequenced as previously described for DNA samples, using the following primers: F: 5′-TCGGAGTCAGAGTCGCAGTG-3′, R: 5′-CAGAGAAGGAGCCAATCATGC-3′, designed respectively on 5′UTR (forward) and on exon 3 (reverse). Electropherograms were analysed with the ChromasPro software (TechnelysiumPty Ltd., Tewantin, QLD, Australia) using the wild-type GLUT1 cDNA sequence (NM_006516) as reference.

## Figures and Tables

**Table 1:** Clinical and biochemical data observed in the GLUT1DS Italian patients. Abbreviations: F, female; M, male; Age, age of diagnosis; Ratio, CSF/blood glucose ratio; NA, not available; +, yes; −, no; MCP, microcephaly; m, months; y, years; sev, severe; mod, moderate; ABS, absence seizure; CFS, complex febrile seizures; DS, dyscognitive seizures; EE, epileptic encephalopathy; ES, epileptic spasms; FS, focal seizures; GTC, generalized tonic-clonic seizures; MAS, myoclonic absence seizures; MS, myoclonic seizures; MD, movement disorder; C, chronic; PED, paroxysmal exertion-induced dyskinesia; PND, paroxysmal non exertion-induced dyskinesia; FD, focal discharges; G, generalized discharges; N, normal; BGS, slow background slowing; KD, ketogenic diet.

Patient	Sporadic/ Familial	Sex	Age	Ratio	MCP	ID	Spasticity	Seizure: Onset/Type	MD: Onset/Type	EEG	KD	Other	Clinical Phenotypes
1 (F1)	Familial	F	NA	0.52	−	nor	−	6y/ABS	-	NA	+		Late-onset classical phenotype
2	Sporadic	M	5m	0.25	−	sev	−	3m/FS, MS	3m/NA	NA	+		Early-onset classical phenotype
3	Sporadic	F	24m	0.37	+	sev	+	3m/FS, ES	-	BGS, FD, EE	+		Early-onset classical phenotype
4 (F2)	Familial	M	10y	0.50	−	nor	−	24m/ABS	7y/PED	N	+		Early-onset classical phenotype
5	Sporadic	F	8y	0.39	−	sev	−	6m/GTC	24m/PD	BGS, FD	+		Early-onset classical phenotype
6	Sporadic	M	11y	0.30	−	sev	−	-	11y/PED	NA	−		Non-classical phenotype
7	Sporadic	F	10y	0.36	−	sev	−	5m/MS	24m/C	BGS, G	−		Early-onset classical phenotype
8	Sporadic	F	7y	0.34	−	sev	−	24m/MAS, DS, ABS	6y/PED	BGS, G, FD	−	stroke-like episode	Early-onset classical phenotype
9 (F3)	Familial	F	7y	0.39	−	mild	+	3y/GTC	NA/no PED	BGS, G, FD	+		Late-onset classical phenotype
10	Sporadic	F	NA	0.39	−	nor	−	12m/DS, GTC, ABS	16m/C	BGS, G, FD	−	stroke-like episode	Early-onset classical phenotype
11 (F4)	Familial	F	7y	0.38	−	sev	−	-	7y/PED	N	−		Non-classical phenotype
12	Sporadic	F	NA	0.50	−	mild	−	3y/ABS	7m/PED	BGS, G	−		Late-onset classical phenotype
13	Sporadic	M	24m	0.39	−	mild	−	15m/MAS	5m/NA	G	+		Early-onset classical phenotype
14	Sporadic	F	11y	0.37	−	sev	+	9m/MAS	-	BGS, G	−		Early-onset classical phenotype
15	Sporadic	F	7y	0.43	−	sev	−	4m/MS, AS	18m/C	G	+		Early-onset classical phenotype
16 (F5)	Familial	F	7y	NA	−	bord	−	6m/ABS	24m/PED, PND	NA	+		Early-onset classical phenotype
17	Sporadic	F	NA	0,42	−	mild	−	24m/ABS	5y/PED	NA	+		Early-onset classical phenotype
18	Sporadic	F	24m	0.31	−	mod	−	20m/ABS	20m/C	G	+		Early-onset classical phenotype
19	Sporadic	M	4y	0.41	+	mild	−	-	-	N	+		Non-classical phenotype
20	Sporadic	M	4y	0.32	+	sev	−	-	24m/PND	N	+		Non-classical phenotype
21	Sporadic	F	12m	0.40	−	mild	−	2m/FS	3m/PND	N	+		Early-onset classical phenotype
22	Sporadic	F	3y	0.38	−	sev	−	3y/GTC	NA/PND	NA	+		Late-onset classical phenotype
23	Sporadic	M	19y	NA	−	NA	NA	8m/NA	-	N	+		Early-onset classical phenotype
24	Sporadic	F	7y	0.38	−	sev	−	3y/ABS, MAS	3m/PED	BGS, G	+		Late-onset classical phenotype
25 (F6)	Familial	M	9m	0.37	−	mod	−	4m/FS	-	FD	+		Early-onset classical phenotype
26	Sporadic	M	NA	0.51	−	sev	−	4y/CFS	14y/PED	FD	−	stroke-like episode	Late-onset classical phenotype
27	Sporadic	M	5y	0.37	−	mild	−	10m/FS, GTC	8y/PED	G	+	stroke-like episode	Early-onset classical phenotype

**Table 2:** *SLC2A1* variants identified in 27 GLUT1DS patients with details on HGVS mutation, exon, location, refSNP, ACMG classification and literature reference for variants already reported. Abbreviations: refSNP, reference SNP; TMH, α-helices transmembrane; IC intracellular domain; VUS, variant of uncertain significance.

Patient	HGVS (Coding)	HGVS (Protein)	Exon/ Intron	Location	RefSNP	ACMG Classification	Literature Data
**1 (F1)**	c.26C > T	p.Thr9Met	2	TMH1	rs1570601100	VUS (PM2 PP2 PP3)	Castellotti et al., 2019 [29]
**2**	c.85_89delAACAC	p.Asn29Trpfs*60	2	TMH1	-	Pathogenic (PVS1 PM2 PM6)	Castellotti et al., 2019 [29]
**3**	c.114+1G > A	-	Intron 2	-	-	Pathogenic (PVS1 PM2 PM6)	Novel
**4 (F2)**	c. 200T > C	p.Leu67Pro	3	TMH2	-	VUS (PM2 PP2 PP3)	Novel
**5**	c.224G > A	p.Gly75Glu	3	TMH2	-	Likely Pathogenic(PM2 PP2 PP3 PM6)	Novel
**6**	c.275+3A > T	-	Intron 3	-	-	VUS (PM2 PP3)	Graziola et al., 2019 [30]
**7**	c.370delC	p.Leu124Trpfs*12	4	TMH4	-	Pathogenic (PVS1 PM2 PM6)	Novel
**8**	c.376C > T	p.Arg126Cys	4	TMH4	rs80359818	Pathogenic (PP5 PM1 PM2 PM5 PM6 PP2 PP3)	Pascual et al., 2002 [3]
**9 (F3)**	c.388G > A	p.Gly130Ser	4	TMH4	rs80359819	Pathogenic (PP5 PM1 PM2 PM5 PM6 PP2 PP3)	Wang et al., 2005 [24]
**10**	c.457C > T	p.Arg153Cys	4	TMH5	-	Pathogenic (PP5 PM1 PM2 PM5 PP2 PP3)	Pascual et al., 2002 [3]
**11 (F4)**	c.493G > A	p.Val165Ile	4	TMH5	rs1057520545	Pathogenic (PP5 PM1 PM2 PM6 PP2)	Urbizu et al., 2010 [9]
**12**	c.497_499delTCG	p.Val166del	4	TMH5	-	Likely Pathogenic (PM1 PM2 PM4 PM6)	Koy et al., 2011 [31]
**13**	c.667C > T	p.Arg223Trp	5	IC2	rs796053248	Pathogenic (PP5 PM2 PM5 PM6 PP2 PP3)	Leen et al., 2010 [5]
**14**	c.745_746delCG	p.Arg249Alafs*131	6	IC2	-	Pathogenic (PVS1 PM2 PM6)	Novel
**15**	c.847C > A	p.Gln283Lys	6	TMH7	-	Likely Pathogenic (PM1 PM2 PM6 PP2 PP3)	Novel
**16 (F5), 17**	c.884C > T	p.Thr295Met	7	TMH7	rs80359823	Pathogenic (PP5 PM2 PM5 PP2 PP3)	Wang et al., 2005 [24]
**18**	c.979_991dup	p.Ala331Glyfs*54	8	TMH8	-	Pathogenic (PVS1 PM2 PM6)	Novel
**19**	c.988C > T	p.Arg330*	8	TMH8	rs80359826	Pathogenic (PVS1 PM2 PM6 PP5)	Wang et al., 2000 [32]
**20**	c.997C > T	p.Arg333Trp	8	TMH8	rs80359825	Pathogenic (PP5 PM1 PM2 PM6 PP2 PP3)	Wang et al., 2000 [32]
**21**	c.1006delC	p.Leu336Cysfs*4	8	TMH9	rs796053271	Pathogenic (PVS1 PM2 PM6 PP5)	Lindy et al., 2018 [33]
**22**	c.1129G > C	p.Ala377Pro	9	TMH10	-	Likely Pathogenic (PM1 PM2 PM6 PP2 PP3)	Novel
**23, 24**	c.1198C > T	p.Arg400Cys	9	TMH10	rs796053263	Pathogenic (PP5 PM1 PM2 PM5 PM6 PP2 PP3)	Liu et al., 2012 [34]
**25 (F6)**	c.1363A > G	p.Thr455Ala	10	IC5	-	VUS (PM2 PP2 PP3)	Novel
**26**	c.1372C > T	p.Arg458Trp	10	IC5	rs13306758	Pathogenic (PP5 PM2 PM5 PM6 PP2 PP3)	Arsov et al., 2012 [35]
**27**	c.1458_1459insT	p.Gly487Trpfs*3	10	IC5	-	Likely Pathogenic (PVS1 PM2 PM6)	Novel

## Data Availability

The data that support the findings will be submitted to gene variant databases (ClinVar, Global Variome shared LOVD) following a 6-month embargo from the date of publication.

## Supplementary Materials

The following supporting information can be downloaded at: https://www.mdpi.com/article/10.3390/ijms232113560/s1.
