# Supplementary material for: Molecular Genetics of GLUT1DS Italian Pediatric Cohort: 10 Novel Disease-Related Variants and Structural Analysis
Source: Int J Mol Sci. 2022 Nov 4;23(21):13560. doi: 10.3390/ijms232113560 (PMC9654628; doi:10.3390/ijms232113560)
Supplement: Supplementary file 1 [file ijms-23-13560-s001.zip › Tables S1 and S2.pdf]

Table S1. Prediction of pathogenicity of the identified inherited missense GLUT1 variant by *in silico* tools.

	Thr9Met	Leu67Pro	Gly130Ser	Val165Ile	Thr295Met	Thr455Ala
BayesDel addAF	Damaging	Damaging	Damaging	Damaging	Damaging	Damaging
BayesDel noAF	Damaging	Damaging	Damaging	Damaging	Damaging	Damaging
DEOGEN2	Damaging	Damaging	Damaging	Damaging	Damaging	Damaging
EIGEN	Pathogenic	Pathogenic	Pathogenic	Pathogenic	Pathogenic	Pathogenic
EIGEN PC	Pathogenic	Pathogenic	Pathogenic	Pathogenic	Pathogenic	Pathogenic
FATHMM	Tolerated	Damaging	Damaging	Tolerated	Damaging	Tolerated
FATHMM-MKL	Pathogenic	Damaging	Damaging	Damaging	Damaging	Damaging
FATHMM-XF	Pathogenic	Damaging	Damaging	Damaging	Damaging	Damaging
LIST-S2	Pathogenic	Damaging	Damaging	Damaging	Damaging	Damaging
LRT	Deleterious	Deleterious	Deleterious	Deleterious	Deleterious	Deleterious
M-CAP	Damaging	Damaging	Damaging	Damaging	Damaging	Damaging
MVP	Pathogenic	Pathogenic	Pathogenic	Pathogenic	Pathogenic	Pathogenic
MutPred	Benign	Pathogenic	Pathogenic	Pathogenic	Pathogenic	Pathogenic
Mutation assessor	High	High	High	Medium	High	High
MutationTaster	Disease causing	Disease causing	Disease causing	Disease causing	Disease causing	Disease causing
PROVEAN	Damaging	Damaging	Damaging	Neutral	Damaging	Damaging
PrimateAI	Damaging	Tolerated	Tolerated	Tolerated	Tolerated	Damaging
SIFT	Damaging	Damaging	Damaging	Damaging	Damaging	Damaging
SIFT4G	Damaging	Damaging	Damaging	Tolerated	Damaging	Damaging

Table S2. Prediction of pathogenicity of the identified novel *de novo* missense GLUT1 variant by *in silico* tools.

	**Gly75Glu**	**Gln283Lys**	**Ala377Pro**
BayesDel addAF	Damaging	Damaging	Damaging
BayesDel noAF	Damaging	Damaging	Damaging
DEOGEN2	Damaging	Damaging	Damaging
EIGEN	Pathogenic	Pathogenic	Pathogenic
EIGEN PC	Pathogenic	Pathogenic	Pathogenic
FATHMM	Damaging	Tolerated	Damaging
FATHMM-MKL	Damaging	Damaging	Damaging
FATHMM-XF	Damaging	Damaging	Damaging
LIST-S2	Damaging	Damaging	Damaging
LRT	Deleterious	Deleterious	Deleterious
M-CAP	Damaging	Damaging	Damaging
MVP	Pathogenic	Pathogenic	Pathogenic
MutPred	Pathogenic	Pathogenic	Pathogenic
Mutation assessor	High	High	High
MutationTaster	Disease causing	Disease causing	Disease causing
PROVEAN	Damaging	Damaging	Damaging
PrimateAI	Damaging	Damaging	Damaging
SIFT	Damaging	Damaging	Damaging
SIFT4G	Damaging	Damaging	Tolerated
