# Supplementary figures and images for: Molecular Genetics of GLUT1DS Italian Pediatric Cohort: 10 Novel Disease-Related Variants and Structural Analysis
Source: Int J Mol Sci. 2022 Nov 4;23(21):13560. doi: 10.3390/ijms232113560 (PMC9654628; doi:10.3390/ijms232113560)

**a**

● N-domain    ● ICH-domain    ● C-domain    ● Exon 4 mutations    ● Nonyl-β-D-Glucoside

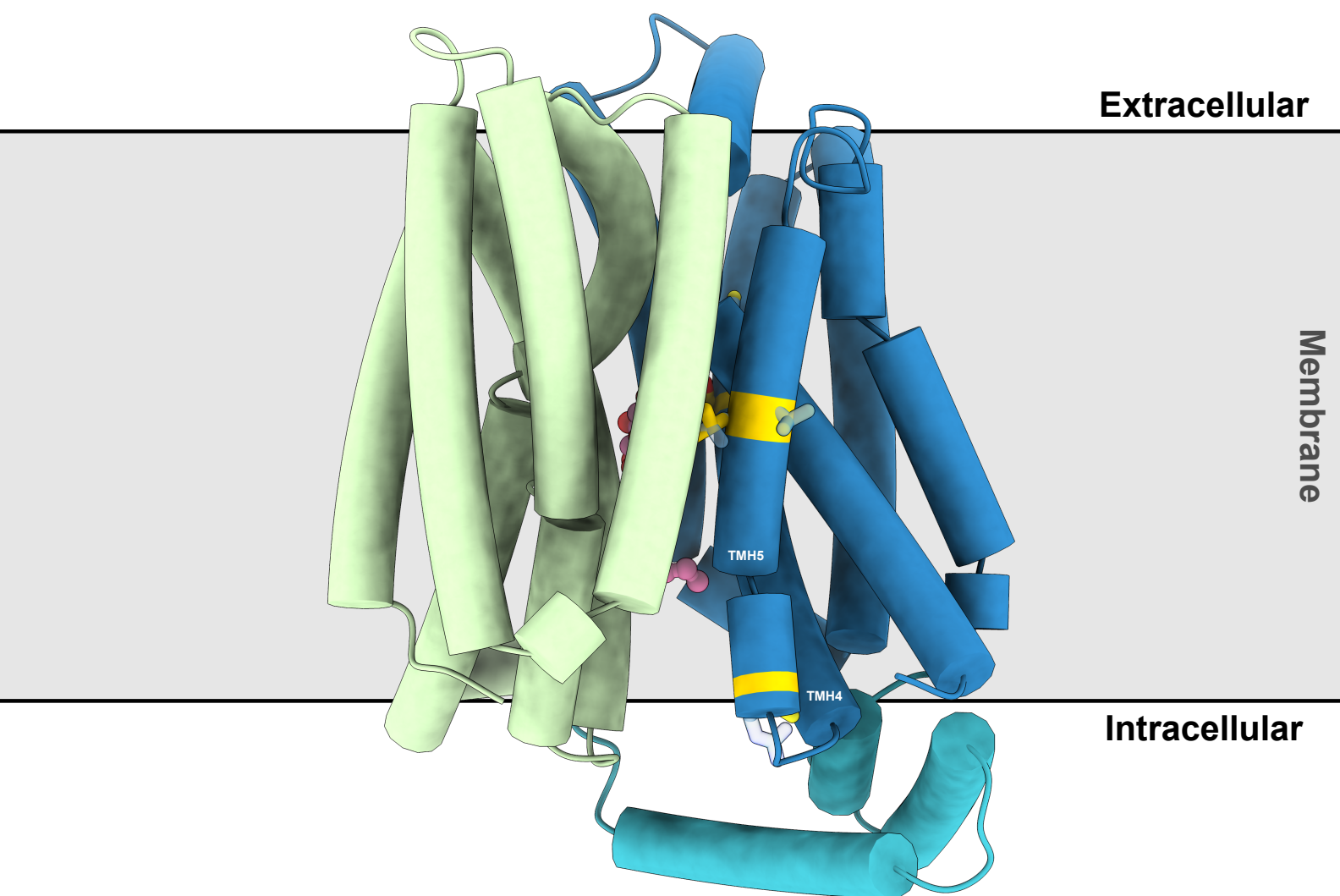**b**

70°

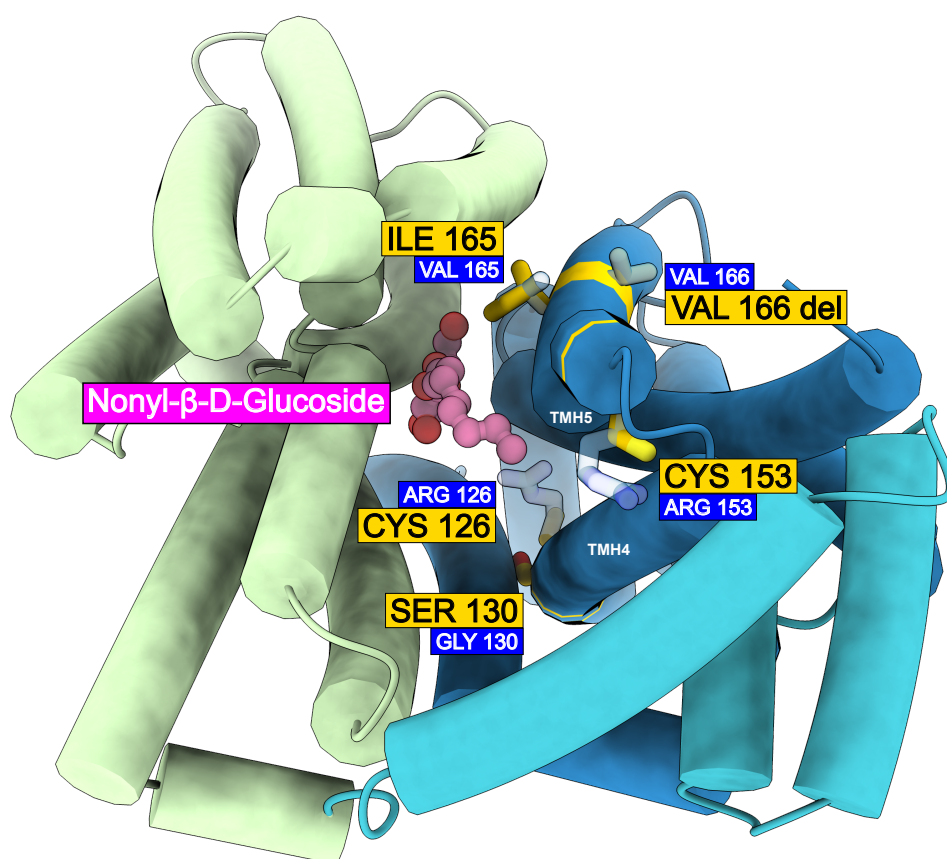

Supplement: Supplementary file 1 [file ijms-23-13560-s001.zip › Figure S1.pdf]
